# The Role of CzcRS Two-Component Systems in the Heavy Metal Resistance of *Pseudomonas putida* X4

**DOI:** 10.3390/ijms160817005

**Published:** 2015-07-27

**Authors:** Pulin Liu, Xi Chen, Qiaoyun Huang, Wenli Chen

**Affiliations:** 1 State Key Laboratory of Agricultural Microbiology, Huazhong Agricultural University, Wuhan 430070, China; E-Mails: liunan3585@163.com (P.L.); chenxi826@163.com (X.C.); 2 Key Laboratory of Arable Land Conservation (Middle and Lower Reaches of Yangtze River), Ministry of Agriculture, College of Resources and Environment, Huazhong Agricultural University, Wuhan 430070, China

**Keywords:** *czcRS*, two-component system, metal resistance, *Pseudomonas putida*

## Abstract

The role of different *czcRS* genes in metal resistance and the cross-link between *czcRS* and *czcCBA* in *Pseudomonas putida* X4 were studied to advance understanding of the mechanisms by which *P. putida* copes with metal stress. Similar to *P. putida* KT2440, two complete *czcRS1* and *czcRS2* two-component systems, as well as a *czcR3* without the corresponding sensing component were amplified in *P. putida* X4. The histidine kinase genes *czcS1* and *czcS2* were inactivated and fused to *lacZ* by homologous recombination. The *lacZ* fusion assay revealed that Cd^2+^ and Zn^2+^ caused a decrease in the transcription of *czcRS1*, whereas Cd^2+^ treatment enhanced the transcription of *czcRS2*. The mutation of different *czcRS*s showed that all *czcRS*s are necessary to facilitate full metal resistance in *P. putida* X4. A putative gene just downstream of *czcR3* is related to metal ion resistance, and its transcription was activated by Zn^2+^. Data from quantitative real-time polymerase chain reaction (qRT-PCR) strongly suggested that *czcRS*s regulate the expression of *czcCBA*, and a cross-link exists between different *czcRS*s.

## 1. Introduction

Heavy metal pollution leads to serious ecological and health problems due to the toxic effects of metal ions and their accumulation throughout the food chain. Even the essential metal ions, Zn^2+^, Cu^2+^, and Co^2+^ could be toxic at high concentrations [1,2,3]. Many microorganisms naturally possess the ability to inhabit metal-polluted areas. An advanced understanding of the mechanisms by which microbes cope with metal stress will facilitate the rational design of strategies for detection and bioremediation of heavy metal-polluted water and soil systems.

To cope with metal stresses, bacteria develop various detoxification mechanisms. The most effective way is the extrusion of excessive metal ions out of the cell by active transport. A number of studies over the past years have been devoted to the protein families that export metal ions. The protein families include P-type ATPases driven by ATP hydrolysis, cation diffusion facilitator family transporters, which act as chemiosmotic ion-proton exchangers, and the resistance nodulation division (RND) family of transporters that mediate proton-driven efflux [4]. Usually, more than one kind of efflux protein is found in metal resistance bacteria [5,6]. CzcCBA is a CBA transporter. This structural gene region encodes outer membrane factors CzcC, membrane fusion protein CzcB, and CzcA protein of the resistance-nodulation-cell division protein family [4]. The transcriptional responses of these exporting genes are controlled by different regulators, including MerR family regulators [7], the ArsR/SmtB family [8], and two-component systems [9,10,11].

Many adaptive bacterial responses to environmental changes are governed by two-component signal transduction systems [12]. In typical two-component systems, environmental variation is first sensed by a histidine sensor kinase capable of autophosphorylation. The phosphoryl group is then transferred to an aspartic acid residue of the response regulator. Usually, the phosphorylated response regulator binds to DNA, resulting in either activation or repression of target genes. Caille *et al.* [9] observed that in *P. aeruginosa* PAO1, Zn^2+^ and Cu^2+^ enhance the expression of *czcRS*. Phosphorylated CzcR then activates the expression of *czcCBA* operon encoding an efflux pump specific for zinc, cadmium, and cobalt. The *copRS* two-component system has been identified as key genes involved in copper resistance [13]. In *Cupriavidus metallidurans* CH34, the regulatory genes of *czcCBA* are arranged in an upstream region consisting of *czcN* and *czcI*, and a downstream region consisting of *czcD*, *czcR*, *czcS* and *czcE* [14]. CzcRS and a periplasmic copper-binding protein designated CzcE [15], exert metal-dependent control of *czcNICBA* expression via regulation of *czcN*p activity [16]. Grosse *et al.* [10] demonstrated that uninduced *C. metallidurans* CH34 with a mutation in *czcS* contains more *czcCBA* message and resumes growth faster when challenged. These pieces of evidence demonstrate the participation of two-component systems in the regulation of metal extrusion.

*P. putida* is a ubiquitous saprophytic bacterium, which has been extensively studied for the biodegradation of organic pollutants [17]. Although the genomes of *P. putida* contain several *czcRS* operons [5], no research has been reported regarding the simultaneous function of different *czcRS*s in the same strain. Our previous study indicated that *P. putida* X4 has a high tolerance and absorption capacity for metal ions [18]. In this study, PCR with primers designed from the homologous sequence in genome sequenced *P. putida* were used to detect the presence of *czcRS* two-component systems and the *czcCBA* operon. Because two *czcRS* two-component systems and one *czcR3* without its corresponding sensor gene were detected, we examined their different roles and how they interacted with the *czcCBA* operon.

## 2. Results

### 2.1. Minimum Inhibitory Concentrations (MICs) of the Wild-Type and Mutant Strains

The MIC values of the wild-type and mutant strains are shown in Table 1. Determination of heavy metal resistance showed that the strain X4 displayed a high level of Cd^2+^, Co^2+^, Cu^2+^, and Zn^2+^ resistance. The functionalities of *czcS1*, *czcS2*, and *czcH* were studied by determining the resistance of gene-deficient strains to different divalent heavy metals. Δ*S1* tended to be sensitive to Cd^2+^, Co^2+^, and Zn^2+^ ions. The MIC of Co^2+^ and Zn^2+^ declined to 0.5 and 2.25 mmol·L^−1^. The *czcS2* mutation decreased the strain’s resistance to Cd^2+^ and Co^2+^. Both *czcS1* and *czcS2* were required for generating full cadmium resistance as the Δ*S1S2* strains with both interrupted *czcS1* and *czcS2* could tolerate only 1.25 mmol·L^−1^ Cd^2+^, which is lower than that tolerated by single gene mutant strains. Small but reproducible repression was observed when Δ*S1* and Δ*S2* were exposed to copper ions. A putative open reading frame (ORF), designated as *czcH* in this study, just downstream of *czcR3*, was identified as necessary to generate full metal tolerance. The resistance of Δ*czcH* to Cd^2+^, Co^2+^, and Zn^2+^ were lower than that of the wild-type strains. The results presented above demonstrate that *czcS1*, *czcS2*, and *czcH* are necessary for the wild-type strain to generate full resistance to Cd^2+^, Co^2+^, Cu^2+^ and Zn^2+^.

**Table 1:** Minimum inhibitory concentrations (MICs) of cadmium, cobalt, copper, and zinc in various derivatives of *P. putida* X4.

Strains	MIC (mmol·L^−1^) ^a^			
	Cd^2+^	Co^2+^	Cu^2+^	Zn^2+^
X4	4.5	4.0	5.0	8.5
X4 with pvlt31	4.5	4.0	5.0	8.5
Δ*S1*	3.0	0.5	4.25	2.25
Δ*S2*	2.0	1.5	4.75	8.5
Δ*czcH*	2.0	1.75	5.0	7.0
Δ*S1S2*	1.25	0.5	4.25	5.0
Δ*S1c*	4.25	3.5	4.5	7.5
Δ*S2c*	4.25	4.0	4.75	8.5
Δ*czcHc*	3.75	3.5	5.0	8.25

^a^ MICs were recorded after 60 h of growth at 30 °C on Tris-buffered medium containing metals salts from 0.5 to 10 mmol·L^−1^, with 0.25 mmol·L^−1^ interval. MIC values are the averages of four determinations.

### 2.2. Genetic Complementation of Mutant Genes

Complementation studies were carried out to confirm that the decreased resistance of strains Δ*S1*, Δ*S2*, and Δ*czcH* to divalent metal ions was caused by the mutation of target genes. For the complementation analysis, the complete ORF of *czcS1*, *czcS2* and *czcH* was cloned into the broad-host-range vector pVLT31, resulting in plasmids pVLTS1, pVLTS2 and pVLTH. Strains Δ*S1c*, Δ*S2c* and Δ*czcHc* were obtained by mating the complementation plasmid into *P. putida* Δ*S1*, Δ*S2* and Δ*czcH*, respectively. The MICs of Δ*S1c*, Δ*S2c* and Δ*czcHc* were tested in Tris-buffered medium with 0.1 mmol·L^−1^ IPTG using the method described above. The complementation data are shown in Table 1. More than 85% of the tolerance to the tested metals was recorded for Δ*S1c* and Δ*czcHc*. The resistance of Δ*S2c* to Co^2+^ and Cd^2+^ were recorded at 100% and 94%, respectively. At the same time, the MICs of control strains did not improve.

### 2.3. Cd^2+^ and Zn^2+^ Repressed the Transcription of czcRS1, Induced the Transcription of czcRS2 and czcH, Respectively

**Figure 1:**
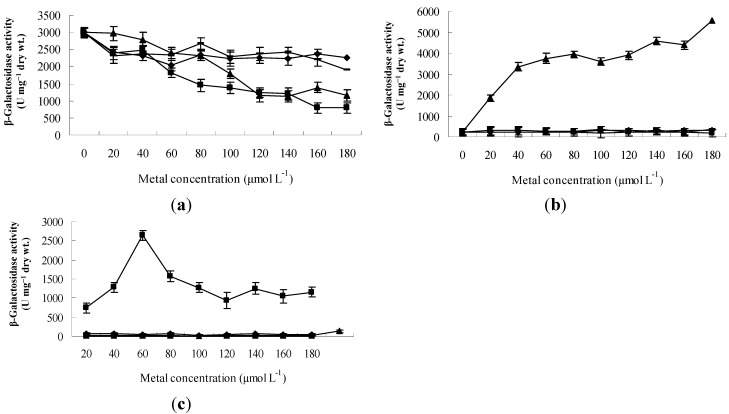
The expression patterns and inducer specificities of *czcS1*, *czcS2*, and *czcH.* LacZ activity were measured in permeabilized cells after induction with different concentrations of Cu^2+^ (◆), Zn^2+^ (■), Cd^2+^ (▲), and Co^2+^ (-) for four hours. Error bars represent the standard deviations of three determinations. (**a**) Response of the promoter *czcRS1p* to different metal ions; (**b**) Response of the promoter *czcRS2p* to different metal ions; and (**c**) Response of the promoter *czcHp* to different metal ions.

**Figure 2:**
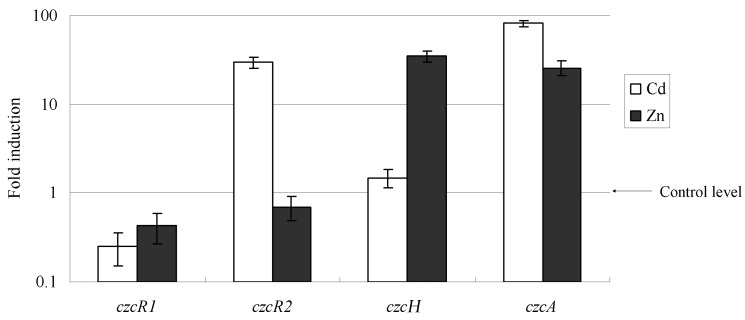
Transcription of *czcR1*, *czcR2*, *czcH*, and *czcA* genes analyzed by qRT-PCR in the wild-type X4 strain grown in the presence of 300 μM Zn^2+^ or Cd^2+^. The amount of mRNA is represented relative to the X4 strain cultivated in the absence of metal ions. Error bars represent the standard deviations of three determinations.

Expression patterns and inducer specificities were determined by measuring the changes of LacZ activity and verified by qRT-PCR. All promoters were tested for their inducibility in the presence of 0–180 μmol·L^−1^ Zn^2+^, Co^2+^, Cu^2+^, and Cd^2+^. The expression patterns and inducer specificities of *czcS1* are shown in Figure 1a. The *czcS1* is transcribed without any metal ions. The transcription of *czcS1* dropped to 40% in the presence of 180 μmol·L^−1^ Zn^2+^, then to 34% after a 180 μmol·L^−1^ Cd^2+^ treatment. Compared with Zn^2+^ and Cd^2+^, Cu^2+^ and Co^2+^ moderately decreased the transcription of *czcS1*. The transcription of *czcS2* was only induced by Cd^2+^; LacZ activity in Δ*S2* increased about 24-fold after exposure to 180 μmol·L^−1^ Cd^2+^ (Figure 1b). The *czcH* gene was significantly induced by Zn^2+^, but was not affected by Co^2+^, Cu^2+^ and Cd^2+^. The highest level of *czcH* transcription was found at 100 μmol·L^−1^ Zn^2+^ and it was decreased when the Zn^2+^ concentration was higher than 100 μmol·L^−1^ (Figure 1c). These observations reveal that transcription from the promoters of *czcS1*, *czcS2* and *czcH* are different, and that the main inducers or repressors are Zn^2+^ and Cd^2+^. qRT-PCR data confirmed the inducibility presented above (Figure 2); in the wild-type strain, addition of 300 μmol·L^−1^ Cd^2+^ or Zn^2+^ to the culture resulted in down-regulation of *czcRS1* and up-regulation of *czcRS2* and *czcH*, respectively (30-, and 35-fold). Besides, the transcription of *czcCBA* was strongly induced by Cd^2+^ (90-fold) and Zn^2+^ (47-fold) to keep the equilibrium of these metal ions in the cells.

### 2.4. The Transcription Variation of Mutant Strains

**Figure 3:**
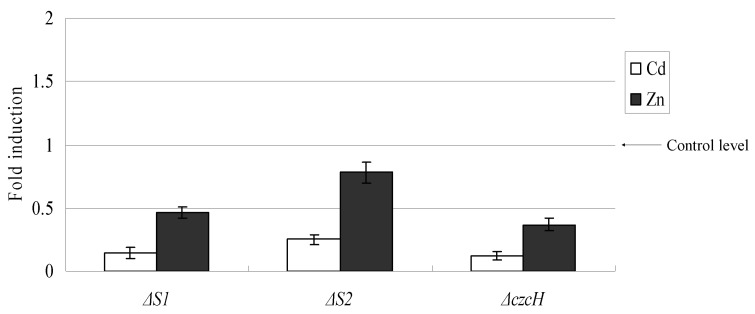
The effects of the mutation in *czcS1*, *czcS2*, and *czcH* on the transcription of *czcA*. Transcription of *czcA* was analyzed by qRT-PCR in the wild-type X4 strain and mutant strains grown in the presence of 300 μM Zn^2+^ or Cd^2+^. The amount of mRNA is represented relative to the wild-type X4 strain cultivated in the same concentration of metal ions. Error bars represent the standard deviations of three determinations.

**Figure 4:**
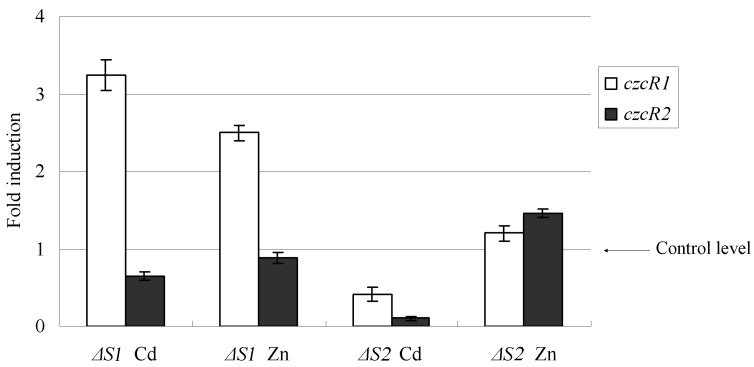
The effects of the mutation in *czcS1* and *czcS2* on the transcription of *czcR1*and *czcR2*. Transcription of *czcR1* and *czcR2* was analyzed by qRT-PCR in the wild-type X4 strain and mutant strains grown with 300 μM Zn^2+^ or Cd^2+^. The amount of mRNA is represented relative to the wild-type X4 strain cultivated in the same concentration of metal ions. Error bars represent the standard deviations of three determinations.

After treatment with Cd^2+^ or Zn^2+^, transcription of *czcA* was determined by qRT-PCR in the wild-type X4 strain and mutant strains. As shown in Figure 3, the mutation of *czcS1* and *czcS2* reduced the mRNA amount of *czcA* to 14%, and 23% that of the wild-type, respectively, in the presence of Cd^2+^. A similar trend was detected upon induction with Zn^2+^. The mutation in *czcH* also caused repressed transcription of the *czcA*. These findings show that all these three genes are required to maintain the expression of *czcA* at a high level. The transcript variation of *czcR1* and *czcR2* is shown in Figure 4. Compared with the wild-type, interruption of *czcS1* enhanced the transcription of *czcR1* by 3.22- and 2.5-fold, in the presence of Cd^2+^ and Zn^2+^, respectively. Mutation of *czcS2* reduced the *czcR1* and *czcR2* mRNA to 40% and 2% of the wild type, while cultured with Cd^2+^. These transcription variations indicated that *czcS1* in wild-type act negatively on the transcription of *czcR1*, and that functional *czcS2* is needed to keep transcription of *czcR1* and *czcR2* at a relatively high level.

### 2.5. Deposition of Strains and Nucleotide Sequences

*P. putida* X4 was deposited in the China Center for Type Culture Collection (CCTCC, http://www.cctcc.org/) with accession number CCTCCM209319. The nucleotide sequences isolated in this study are available in the NCBI Genbank database: *czcR1* (HQ676126), *czcR2* (HQ676125), *czcS1* (HQ676127), *czcS2* (HQ676128), *czcA* (HQ676129), czcH (*dsgR3*, HQ676130), *czcR3* (HQ676124). The similarities of deduced amino acid sequences of *czcR* and *czcS* genes are available in Table S1.

## 3. Discussion

In the present study, we demonstrate the roles played by the *czcRS* two-component systems in the control of *czcCBA* transcription as well as their different inducibility. To our knowledge, this is the first report on the functional difference of *czcRS* two-component systems in *P. putida*. Our results clearly show that all *czcRS* two-component systems are needed for *P. putida* X4 to generate full metal resistance. The reductions in MICs of Zn^2+^, Co^2+^, and Cd^2+^ in the *czcS* mutant strains were less than eight-fold. It cannot be excluded that *czcCBA* is also induced by other regulators, or that other transporters present in X4 may contribute to the intrinsic resistance of this organism to heavy metals. The amplified *czcA* in this study which is induced by Zn^2+^, and Cd^2+^ in wild type revealed a >99% nucleotide identity with the *czcCBA1* operon in *P. putida* KT2400. The *czcCBA1* operon in *P. putida* KT2440 plays a major role in generating metal resistance to Zn^2^^+^, Cd^2^^+^, and possibly Pb^2^^+^ [6]. The *czcCBA* found in *C. metallidurans* CH34 confers resistance to Zn^2+^, Co^2+^, and Cd^2+^ [10]. Similar systems have been found in *P.* *aeruginosa* PAO1 and *P.* *aeruginosa* CMG103 [9,19].

**Figure 5:**
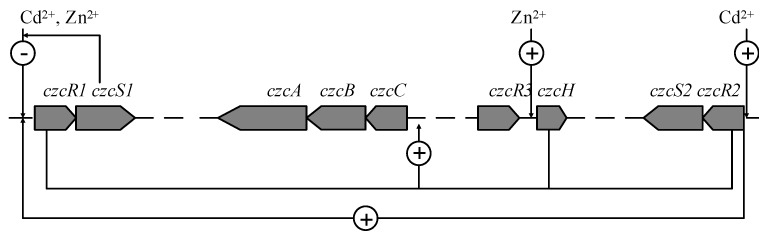
Model representing the cross-interaction among Zn^2+^, Cd^2+^, *czcRS*, and *czcA*. Based on the data from the LacZ fusion assay and qRT-PCR, the cross-talk among Zn^2+^, Cd^2+^, *czcRS*, and *czcA* was depicted. Zn^2+^ and Cd^2+^ repressed the transcription of *czcRS1*, and induced the transcription of *czcRS2* and *czcH*, respectively. The *czcR*s and *czcH* gene could induce *czcCBA* transcription, leading to resistance to cobalt, zinc, and cadmium. Data from qRT-PCR also revealed a cross-interaction between *czcRS2* and *czcRS1*.

Plasmid pVIK112 was constructed by Kalogeraki *et al.* [20] to simultaneously disrupt and create fusions to target genes of diverse bacteria. The sequence of the fragment amplified in this study and the complete genome analysis of *P. putida* KT2440 [5] revealed that the *czcRS* in *P. putida* form separate operons and *czcSs* is located at the end of the operons. Therefore, a minimal polar effect was generated after single cross-over with pVIK112. The remarkable increase in metal resistance in the complementation strains supports this idea. Combining the data from the β-galactosidase activity assay and the transcription variation of *czcR*s and *czcCBA*, the cross-interaction pathway among Zn^2+^, Cd^2+^, *czcRS* and *czcCBA* was depicted (Figure 5). The control of *czcCBA* expression through *czcRS* has been studied in *P. aeruginosa* and *C.* *metallidurans* [9,10,11]. Compared with previous studies, the regulation of *czcCBA* in *P. putida* was different. *P. putida* evolved two *czcRS* operons. Simultaneously, other regulation genes, such as *czcN* and *czcI* were not found in the *P. putida* sequenced genome. The expression of the CzcCBA efflux pump was induced by two *czcRS* operons with different inducibilities. The *czcH* gene also influenced the transcription of *czcCBA*. The function of *czcRS1*, *czcRS2* and *czcH* in *P. putida* X4 may be complementary. The transcription of *czcRS1* was inhibited in the presence of Zn^2+^ and Cd^2+^, which may caused the reduced transcription of *czcCBA*. However, a high level of efflux pump CzcCBA expression is essential for coping with high concentrations of metal ions (Figure 2). The low expression trend of *czcCBA* resulting from the CzcR1 may be compensated for by the induction effect caused by *czcRS2* and *czcH*, which were induced with Cd^2+^ and Zn^2+^, respectively. Complete *czcRS1* negatively feedbacks on its expression, which creates a common regulating pathway in prokaryotes for maintaining the expression of transcription factors at appropriate levels [21]. Complete *czcRS2* has a positive effect on the transcription of itself. This positive autoregulation loop could allow the cells to respond rapidly to the presence of small amounts of heavy metals in the environment [11].

In conclusion, there was a clear distinction between the different *czcRS*s. *czcRS1* is constitutively expressed and possibly acts as a housekeeping resistance mechanism against Zn^2+^, Co^2+^, and Cd^2+^ in *P. putida* X4. The transcription of *czcRS1* was repressed by Cd^2+^ and Zn^2+^, whereas the transcription of *czcRS2* was induced only by Cd^2+^. Both *czcS1* and *czcS2* act positively on the transcription of *czcA*. Although *czcH* plays an important role in the metal resistance of *P. putida* X4, the underlying mechanism merits further studies. Different *czcRS* genes, along with other regulators, are needed to make precise adjustments to the gene expression of *P.* *putida* to adapt to changes in metal ions in the environment.

## 4. Materials and Methods

### 4.1. Bacterial Strains, Plasmids, and Growth Conditions

Detailed information regarding the bacterial strains and plasmids are listed in Table 2. *P. putida* X4 was isolated from the waste soil around the Academy of Hubei Agricultural Sciences, Wuhan, China [22]. For construction and maintenance of plasmids, *Escherichia coli* strains DH5α and C118λ*pir* were used.

**Table 2:** Bacterial strains and plasmids used in this study.

		Relevant Characteristics ^a^	Reference
**Strains**	*E. coli*		
	DH5α	*supE*44 Δ*lacU*169 (Ф80*dlacZ*Δ*M*15) *recA*1 *endA*1 *hsdR*17 *thi*-1 *gyrA*96 *relA*1	Invitrogen
	C118λ*pir*	Δ(*ara-leu*) *araD* Δ*lacX*74 *galE* *galK* *phoA*20 *thi*-1 *rpsE* *rpoB* *argE* (Am) *recA*1 λ*pir*	[23]
	MM294	*endA thiA hsdR*17 *supE*44	[24]
	*P. putida*		
	X4	Ap^r^, Co^r^, Zn^r^, Cd^r^, Cu^r^, wild-type	[22]
	Δ*S1*	X4Δ*czcS1*	This study
	Δ*S1c*	X4Δ*czcS1*, with plasmid pVLTS1	This study
	Δ*S2*	X4Δ*czcS2*	This study
	Δ*S2c*	X4Δ*czcS2*, with plasmid pVLTS2	This study
	Δ*czcH*	X4Δ*czcH*	This study
	Δ*czcHc*	X4Δ*czcH*, with plasmid pVLTH	This study
	Δ*S1S2*	X4Δ*czcS1czcS2*	This study
**Plasmid**	pRK2073	RK2 helper plasmid, Spe^r^	[24]
	pTA2	Cloning vector, Amp^r^	TOYOBO
	pVIK112	*LacZYA*, Km^r^, suicide vector	[20]
	pVIks1	*S1* fragment in pVIK112	This study
	pVIKs2	*S2* fragment in pVIK112	This study
	pVIKH	*czcH* fragment in pVIK112	This study
	pRRT	Contains a *Tet^r^* gene instead of *Km^r^* cassette	This study
	pRRTS2	*S2* fragment in pRRT	This study
	pVLT31	*lacI*, Tet^r^	[25]
	pVLTS1	pVLT31 with *S1* ORF	This study
	pVLTS2	pVLT31 with *S2* ORF	This study
	pVLTH	pVLT31 with *czcH* ORF	This study

^a^ Co^r^, Zn^r^, Cd^r^, and Cu^r^ indicate resistance to cobalt, zinc, cadmium, and copper, respectively; *Amp^r^*, *Km^r^*, *Tet^r^*, and *Spe^r^* stand for resistance to ampicillin, kanamycin, tetracycline, and spectinomycin, respectively.

Bacteria were routinely grown in Luria–Bertani medium at 37 °C (*E. coli*) or 30 °C (*P. putida*). When required, antibiotics were added at the following final concentrations: for *E. coli*, 100 µg/mL ampicillin, 50 μg/mL kanamycin, and 12.5 μg/mL tetracycline; and for *P. putida*, 50 μg/mL kanamycin and 12.5 μg/mL tetracycline. Tris-buffered medium [26] containing 0.1% glucose was used for testing metal resistance and promoter inducibility. Analytical grade reagents CuSO_4_·5H_2_O, CdCl_2_·H_2_O, ZnCl_2_, and CoCl_2_·6H_2_O were prepared into 1.0 M stock solutions and sterilized by filtration.

### 4.2. Determination of MIC Values

The minimum inhibitory concentrations (MICs) of trace metals were determined as previously described [26]. Briefly, bacteria cultures were spread onto the Tris-buffered medium containing metal salts from 0.5 to 10 mmol·L^−1^ (0.25 mmol·L^−1^ interval). The MICs were defined as the lowest concentration of metal salts at which no CFU were observed after 60 h of incubation at 30 °C.

### 4.3. DNA Manipulation

All primer pairs used in this study were shown in Table 3. Axygene mini-prep, PCR purification, and gel purification kits (Axygene, Union City, CA, USA) were chosen for DNA separation and purification. PCRs were carried out using Taq DNA polymerase. The plasmids were constructed using standard recombinant DNA techniques and introduced into *E. coli* by transformation. Plasmids derived from pVIK112 [20] were introduced into *P. putida* by triparental conjugation. The helper strain was *E.* *coli* MM294 with the helper plasmid pRK2073 [24]. The essential DNA fragment was verified by DNA sequencing.

**Table 3:** Primers used in this study.

Purpose	Primer Pair	Sequence (5ʹ-3ʹ) ^a^	Product Length (bp)
For homologous recombination	S1	gag cag acc tgg aag taa aga	1113
		ggt aga acc gct caa aca a	
	S2	cgt agg cta tgt act tga ggc g	920
		tgt cgt tga tga tgc ggt tg	
	czcH	cac agg gca ttc agg gac caa cgc acg gga taa gag	496
		gcc cgt tgc acc aca gat	
For qRT-PCR	Qr1	aca acg gtg tag atg ctc tgc	121
		cgg ctg gtc tta cgg atg g	
	Qr2	gcc gca acg acc agc aac	144
		gac gca tca gca ggt gta gc	
	Qr3	atg atg ctg acg gcg aga ag	162
		gcg aat gac ctc tac gga tgc	
	QczcA	cca ctg agc acg acc aag g	128
		aag gtg aag gaa gag gaa ggc	
	QrpsL	ctg cgt aaa gta tgc cgt gtg	174
		gcc cga agt atc cag aga gc	
For complementation experiment	CS1	cgg ggt acc taa gaa gga gat ata cca tga ggc cat tca gcc tgg	1455
		cta gtc tag att aag cgg cgg tca ttg c	
	CS2	cgg ggt acc taa gaa gga gat ata ccttg aaa aac gcc agc ctg tc	1419
		cta gtc tag atc act cgg cag gaa aca cca	
	CczcH	cgg ggt acc taa gaa gga gat ata cca tga ggt ata gca ttg att atc agc a	360
		cta gtc tag att ata aga agg cga gcg ag	
For tetracycline resistance gene	Tet	cga cct gca gaa aat agg cgt atc acg agg	1560
		cag cct gca gtc tgc taa cca gta agg caa cga gg	

^a^ underlined are restriction sites.

### 4.4. Homologous Recombination for Construction of czcS Mutants and lacZ Fusion Reporter Strains

The suicide fusion vector pVIK112 was selected for the mutation and construction of a *lacZ* fusion reporter system. The plasmid pVIKS1 containing a *czcS1* fragment was constructed as follows: the *czcS1* fragment was cloned into pTA2 and its orientation was confirmed by sequencing. After digestion with *Xba*I and *Kpn*I, the restriction fragment was cloned into pVIK112 to create pVIKS1. The same procedure was then employed to create plasmids pVIKS2 and pVIKH. These plasmids were introduced into *P. putida* X4 by triparental conjugation. To generate double *czcS* mutation strain Δ*S1S2*, another suicide plasmid pRRT was constructed by replacing *lacZYA* and the kanamycin resistance gene cassette in pVIK112 into a tetracycline resistance gene by *Pst*I digestion. After ligating with *czcS2* restriction fragment from pTA2, the plasmid was applied to generate double mutation based on the Δ*S1*.

### 4.5. β-Galactosidase Activity Assay

The activity of β-galactosidase was measured according to the method by Nies [27] using *o*-nitrophenyl-d-galactoside (4 mg/mL) as the substrate. The unit 1 U was defined as the activity that forms 1 nmol of *o*-nitrophenol per minute at 30 °C.

### 4.6. Complementation Experiment

Complementation experiments were carried out to confirm that the phenotype variation is caused by the *czcS* mutant. Two *czcS* and the *czcH* open reading frames (ORFs) were amplified using the total DNA from *P. putida* X4 as the template. After digestion with *Xba*I and *Kpn*I, the amplified fragments were ligated into the broad-host-range expression vector pVLT31, which contains the *lacI* and *tac* promoter [25], yielding pVLTS1, pVLTS2, and pVLTH. The yielded plasmids were mated into mutant strains Δ*S1*, Δ*S2*, and Δ*czcH* respectively via conjugation. The MICs of the different transconjugants were checked as described above with IPTG induction. Control strains were constructed by transformed pVLT31 into Δ*S1*, Δ*S2*, *and* Δ*czcH*.

### 4.7. RNA Isolation and Quantitative Real-Time Polymerase Chain Reaction (qRT-PCR) Analysis

After Cd^2+^ or Zn^2+^ treatment, the total RNA for qRT-PCR was extracted using the RNA bacteria protect solution and the RNeasy kit (Qiagen, Hilden, Germany) according to the manufacturer’s instructions. The residual DNA was eliminated by treatment with 20 U RQ1 RNase-free DNase (Promega, Madison, WI, USA) followed by phenol-chloroform extraction. The RNA was precipitated with ethanol and resuspended in RNase-free water. For cDNA synthesis, 3 μg of RNA was reverse transcribed using random hexamer primers and MLV reverse transcriptase (Promega) according to the supplier’s instructions. Reverse transcriptase was inactivated by incubation at 70 °C for 15 min and the obtained cDNAs were stored at −20 °C until utilization. The cDNAs were quantitatively measured with a Bio-Rad iCycler machine (Bio-Rad, Berkeley, CA, USA) using a Sybr Green Quantitect kit (Qiagen). The primer pairs used for qRT-PCR were designed using the Primer3 [28] program. To check for contaminated DNA, control reactions without reverse transcriptase were analyzed using *rpsL* primer set. The pure RNA samples were obtained when no amplification of the non-template control was detected. The cDNA samples were diluted 10-fold and 1 μL of the dilution served as the PCR template, which was performed in duplicate for each gene and sample. To offset the difference in the amount of cDNAs, the ribosomal *rpsL* gene was chosen as the reference gene. Results are presented as the ratios of gene expression between the target gene (target) and the reference gene (*rpsL*), which were obtained according to the following equation: ratio = (*E*_target gene_)^Δ*C*t target (^^wild type − test strain)^/(*E_rpsL_*)^Δ*C*t *rpsL* (^^wild type − test strain)^ [29], where *E* is the real-time PCR efficiency of a given gene and *C*t is the crossing point of the amplification curve with the threshold. An effect on gene transcription was considered significant when the corresponding ratios were ≥2.0 or ≤0.5.

## Supplementary Materials

Supplementary materials can be found at http://www.mdpi.com/1422-0067/16/08/17005/s1.
