# Supplementary material for: The Role of CzcRS Two-Component Systems in the Heavy Metal Resistance of Pseudomonas putida X4
Source: Int J Mol Sci. 2015 Jul 27;16(8):17005–17. doi: 10.3390/ijms160817005 (PMC4581181; doi:10.3390/ijms160817005)
Supplement: Supplementary file 1 [file ijms-16-17005-s001.pdf]

Supplementary Information

Table S1. The sequence similarity between different *czcR* and *czcS* genes.

	Sequence Similarity				
**Genes**	***czcR*1**	***czcR*2**	***czcR*3**	***czcS*1**	***czcS*2**
***czcR*1**	100%	–	–	–	–
***czcR*2**	69%	100%	–	–	–
***czcR*3**	67%	73%	100%	–	–
***czcS*1**	–	–	–	100%	–
***czcS*2**	–	–	–	47%	100%
